# Semi‐Automated Analysis of Lymph Node Immune and Fibroblast Architecture From Immunofluorescent Images

**DOI:** 10.1002/cpz1.70447

**Published:** 2026-08-20

**Authors:** Kassandra Vezyrgianni, Stephen Rothery, Alice E. Denton

**Affiliations:** ^1^ Department of Immunology and Inflammation Imperial College London London United Kingdom; ^2^ Facility for Imaging by Light Microscopy (FILM) Imperial College London London United Kingdom

**Keywords:** aging immunology, Fiji Macros, image analysis, immunofluorescence, lymph node

## Abstract

Tissues are highly ordered structures, with cellular localization essential to the optimal function of the tissue. In lymph nodes the spatial arrangement of immune cells and their associated with stromal cell architecture is essential for the generation of optimal immune responses. Circumstances such as aging and chronic disease can alter lymph node architecture in a manner that impacts immune function. Immunologists have developed many tools for analyzing the cellular content of lymphoid tissue, with flow cytometry a central workhorse in the field. While flow cytometry is a powerful high‐throughput tool for analyzing multiple cell types in a tissue, it cannot assess where those cells are located. Imaging, on the other hand, is relatively low throughput, with objective image analysis often presenting a significant hurdle for many immunologists. Here we describe two analysis tools built in Fiji (ImageJ) that semi‐automate the analysis of lymph node spatial architecture, can be adapted to different antibody staining panels, and are straightforward to implement with little computational experience. © 2026 The Author(s). *Current Protocols* published by Wiley Periodicals LLC.

**Basic Protocol 1**: Quantifying changes in lymph node regional architecture

**Alternate Protocol**: Adapting KVA1 for use on spleen tissue sections

**Basic Protocol 2**: Measuring follicular dendritic cell network area within individual B cell follicles

## INTRODUCTION

Lymph nodes act as a sieve for potential invading agents in recirculating lymph; the lymph returns from the periphery through lymph nodes via lymphatics, which surround the cortex and paracortex, and empties into the medullary sinuses to return to circulation (Girard et al., 2012). Within the cortex and paracortex lymphocytes localize to specific regions: T cells within the T cell zones; and B cells within the follicle. The restriction of lymphocytes to specific tissue regions is largely controlled by fibroblasts that secrete chemokines that direct T and B cell homing (Cinti & Denton, 2021). The follicle is the site in which antibody responses are generated; it is supported by follicular dendritic cells (FDCs), a highly specialized fibroblast that displays antigens to B cells to initiate and sustain immune responses (Allen & Cyster, 2008). The organization of lymphocytes within lymph nodes, as directed by fibroblasts, is essential for the optimal functioning of the immune response. For example, when fibroblasts are impaired in their ability to fully mature, the immune response is reduced (Chai et al., 2013). More recent studies have demonstrated that regional zoning within the lymph node is essential for its function. For example, crosstalk between dendritic cells and fibroblasts dictates development of chemokine gradients that dictate the position of CD8+ T cells (Alouche et al., 2026), while disrupting the integrity of the fibroblast network impacts the generation of adaptive immunity (Makris et al., 2025). Finally, conditions in which fibroblasts and lymph node architecture are disrupted, such as chronic infection/inflammation and aging, are associated with impaired immune responses (Cinti & Denton, 2021). Thus, the ability to analyze the spatial architecture of lymphoid tissues facilitates understanding its impact on immune responses.

Several computational tools have been used to analyze aspects of lymphoid tissue architecture. Stretching of the fibroblast network, which accommodates incoming immune cells prior to fibroblast expansion, has been measured with a custom MATLAB script that assesses the gaps between FRCs in 2D immunofluorescence images (Acton et al., 2014); larger gaps indicate lymphoid fibroblasts are further apart. This tool has also been used to show biomechanical tension of lymphoid fibroblasts impacts immune responses (Horsnell et al., 2022) and how lymphoid fibroblast development impacts the fibroblast network structure (Horsnell et al., 2024). Other methods have analyzed the topology of the lymphoid fibroblast network in 3D (Novkovic et al., 2016, 2017, 2020) using Imaris to annotate interconnection between fibroblasts, which is then analyzed in R studio to understand the structure of the network using a range of statistical methods. These tools, while powerful, have a certain entry barrier or require proprietary software for analysis. In this article, we describe a simple, yet customizable, tool to analyze lymph node architecture in immunofluorescence images using Fiji (ImageJ).

Fiji (Schindelin et al., 2012) is an entirely open‐source tool that has become the first choice for many scientists aiming to perform rudimentary or more advanced analyses. Many researchers create their own plug‐ins, or macros, to answer specific research questions or make image processing more efficient. However, there often is a high barrier of entry for both students and researchers who are primarily skilled in “wet lab” techniques and lack computational analysis experience. Moreover, the user interfaces of computational tools can appear complicated and/or confusing to image analysis novices (North, 2006). Here we describe two simple, adaptable, Fiji macros that automatically analyze the size of lymph nodes and specific immune cell regions within the lymph node; and the FDC networks within specific cellular regions.

## Basic Protocol 1 QUANTIFYING CHANGES IN LYMPH NODE REGIONAL ARCHITECTURE

This protocol uses KVA1 to measure the size of the whole lymph node, and calculate the area of specific stained regions (in this case the T cell zone, B cell follicles, and the lymphatic endothelium) in a semi‐automated manner.

### Necessary Resources

#### Hardware

A computer with a suitable 64‐bit operating system that meets Fiji's minimum installation requirements, with a ≥16 GB RAM and an appropriately fast CPU

##### Software

The latest version of FIJI or ImageJ (Stossi & Singh, 2023)

*Please ensure that Fiji is updated regularly*.

KVA1 macro script available at: https://github.com/DentonLab/LN‐Architecture‐Macros.git

##### Files

Tile‐scan microscopy images of appropriately stained lymph node sections (Fra‐Bido et al., 2021; Rojas‐Henríquez et al., 2025; Shihan et al., 2021)

#### Image pre‐processing

1 Import the files into Fiji as colorized images of the highest resolution option available and split into separate channels.
2 Adjust the brightness and contrast of each channel was to remove background fluorescence and ensure good definition of fine details (Image > Adjust > Brightness & Contrast).
  To keep the analysis as unbiased and internally consistent as possible, maintain the brightness and contrast settings approximately the same across all tissues of the same group.
3 Merge the three channels into an overlaid image (Image > Color > Merge channels).
4 Save pre‐processed images saved as new TIFF files for macro analysis.

#### Operating the KV1 macro (Video 1)

**Figure 1:**
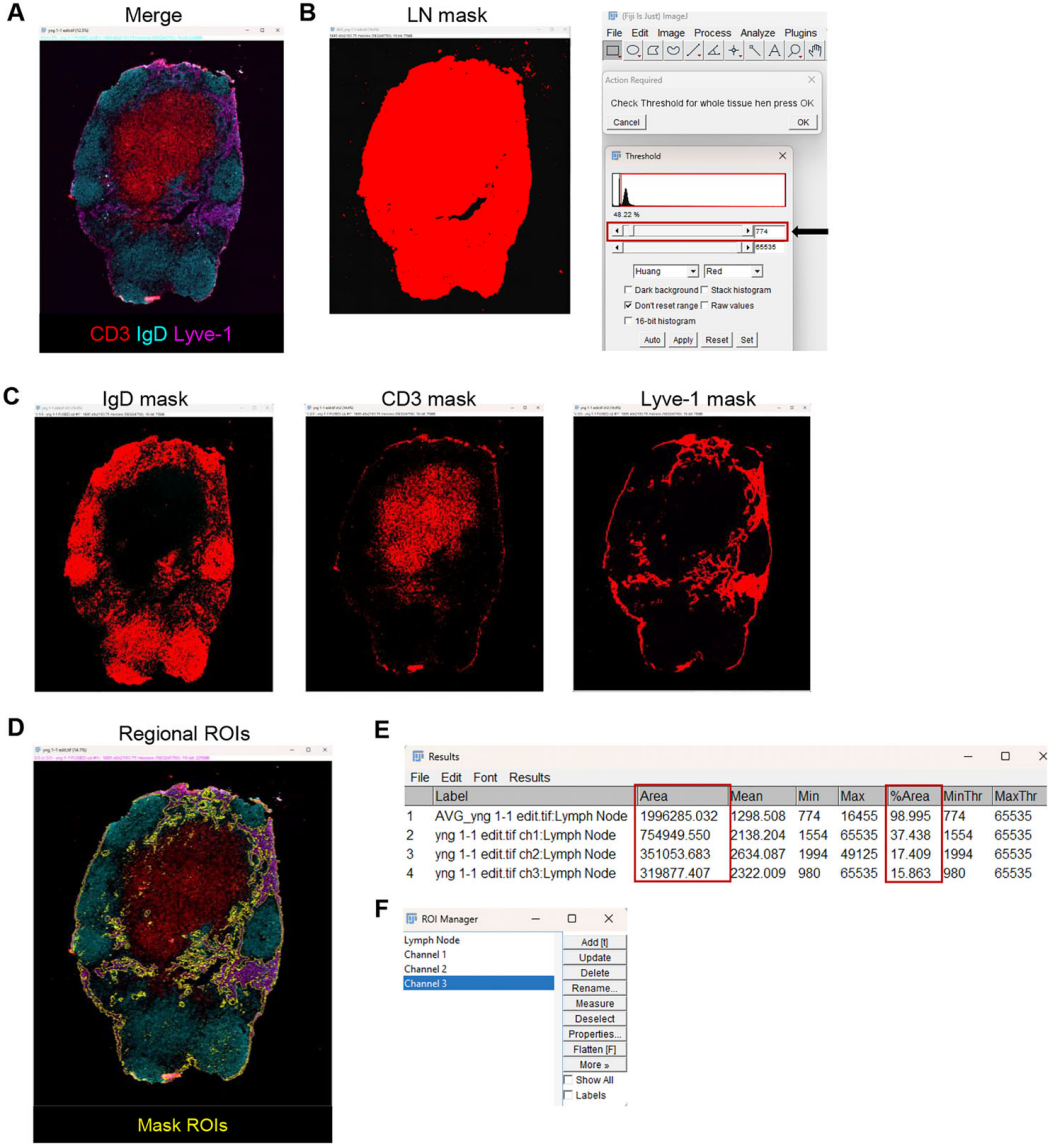
Step‐by‐step visualization of Basic Protocol 1. (**A**) Pre‐processed tiled microscopy image of IF‐stained cervical lymph node using the 20× objective lens, opened in Fiji. Follicles identified by IgD staining in cyan, T cell zone by CD3 staining in red and lymphatic endothelial cells (LECs) by Lyve‐1 staining in magenta. (**B**) Basic Protocol 1, step 10: manual thresholding to identify whole tissue. (**C**) Basic Protocol 1, step 12: examples of manual thresholding to identify the follicles (Channel 1 “IgD mask,” left), the T cell zone (Channel 2 “CD3 mask,” center), and the lymphatics (Channel 3 “Lyve‐1 mask,” right). (**D**) Example of ROI mask for lymphatics overlaid onto the analyzed lymph node. (**E**) Analysis table with key measurements highlighted in red. (**F**) ROI manager output of (**D**). Also see Video 1.

5 Open the pre‐processed TIFF images of whole lymph nodes (Fig. 1A).

**Video 1:** Using the KVA1 macro. 00:06 to 00:33: Adjusting threshold settings to create whole tissue mask. 00:34 to 01:00: Adjusting threshold settings to detect B cell follicles (Channel 1). 01:01 to 01:21: Adjusting threshold settings to detect T cell zone (Channel 2). 01:22 to 01:46: Adjusting threshold settings to detect LEC network area (Channel 3). 01:47 to 02:00: Demonstrating the results table. 02:01 to 02:18: Demonstrating the individual region masks on the ROI manager.

6 To start the KVA1 macro, simply drag‐and‐drop the script file from its downloaded location onto the Fiji toolbar; this will open a new window showing the macro script. Click on the image of interest, then press “run”.
7 KVA1 will automatically run the “Set Measurements” command (Analyze > Set Measurements…), selecting “Area,”, “Mean gray value,” “Min & Max gray value,” and “Area Fraction,” and setting the number of decimal places to 3 and the redirect value to “None.”
8 KVA1 automatically clears any previous selections and ROIs from the ROI manager and any previous measurement results, then retrieves the image name, dimensions, and voxel size.
9 To create a mask of the whole lymph node, KVA1 first runs the “Z project” command with average intensity (Image > Stacks > Z Project…) and “Gaussian Blur” (Process > Filter > Gaussian blur…) with a sigma = 5 to segment and smoothen the whole lymph node area, before auto‐thresholding (Image > Adjust > Threshold) with the “Huang dark” setting.
  It should be noted that the macro does not require a z‐stack image; rather, the “Z Project…” command here is used to produce a single image of all the channels combined, so that a binary mask can be created of the whole tissue.
10 A new window then prompts the user to manually check the thresholding values to ensure the whole tissue is covered, without overexposing any background signal or debris such as fat or vessels that are outside the lymph node (Fig. 1B). Once satisfied, press “OK” and KVA1 will run the “Analyze Particles” command (Analyze > Analyze Particles…) to create an ROI of the entire lymph node, add it to the ROI manager, where the ROI is renamed (Lymph Node) and its area is measured (Fig. 1D‐F).
11 To measure each individual compartment, KVA1 first duplicates the image, isolating the stain in each channel separately, working sequentially through channels in numerical order (1, 2, then 3).
12 For each channel, the user is prompted to manually check the thresholding to ensure that the staining is clearly defined and background is excluded (Fig. 1C), as for the whole lymph node outline (Fig. 1B). Press “OK” and KVA1 will create an ROI for each channel, which is renamed (Channel 1/2/3), and measured (Fig. 1D‐F).
13 The process is repeated for the remaining two channels, and the results of all four measurements (whole lymph node and each channel stain) are presented in the Results window (Fig. 1E). The results window calculates cross‐sectional area of each stain (“Area,” in µm^2^) and then the percentage of the whole lymph node area that each channel occupies (“%Area”) (Fig. 1E).
14 Export the measurements into data processing software, e.g., Excel or GraphPad Prism, for collation, organization, and statistical analysis.

## ADAPTING KVA1 FOR USE ON SPLEEN TISSUE SECTIONS

KVA1 can also be used to analyze the splenic white pulp, with modifications outlined in this protocol.

### Necessary Resources

See Basic Protocol 1

1 If the image to be analyzed is a tile scan of a spleen cross‐section containing multiple white pulp regions, individual white pulps should be first selected using a rectangular ROI and duplicated as separate images. It is recommended that ROIs closely crop the white pulp to minimize any background or non‐specific staining from the red pulp.
2 Before running KVA1, the user should amend the script to account for the smaller size of WPs compared to lymph nodes (LNs). In line 17 of the script, change the parameters of the “Analyze Particles” command to “size = 40000‐Infinity” (for lymph node “size = 100000‐Infinity”) to ensure smaller white pulp regions are not excluded.
3 KVA1 can then be run without any other modifications, as per Basic Protocol 1.

## Basic Protocol 2 MEASURING FDC NETWORK AREA WITHIN INDIVIDUAL B CELL FOLLICLES

This protocol uses KVA4 to isolate individual B cell follicles and measure the size of each follicle and its FDC network in a semi‐automated manner.

### Necessary Resources

#### Hardware

A computer with a suitable 64‐bit operating system that meets Fiji's minimum installation requirements, with a ≥16 GB RAM and an appropriately fast CPU

##### Software

The latest version of FIJI or ImageJ (Stossi & Singh, 2023)

*Please ensure that Fiji is updated regularly*.

KVA4 macro script available at: https://github.com/DentonLab/LN‐Architecture‐Macros.git

##### Files

Tile‐scan microscopy images of appropriately stained lymph node sections (Fra‐Bido et al., 2021; Rojas‐Henríquez et al., 2025; Shihan et al., 2021)

#### Operating the KV4 Macro (Video 2)

**Figure 2:**
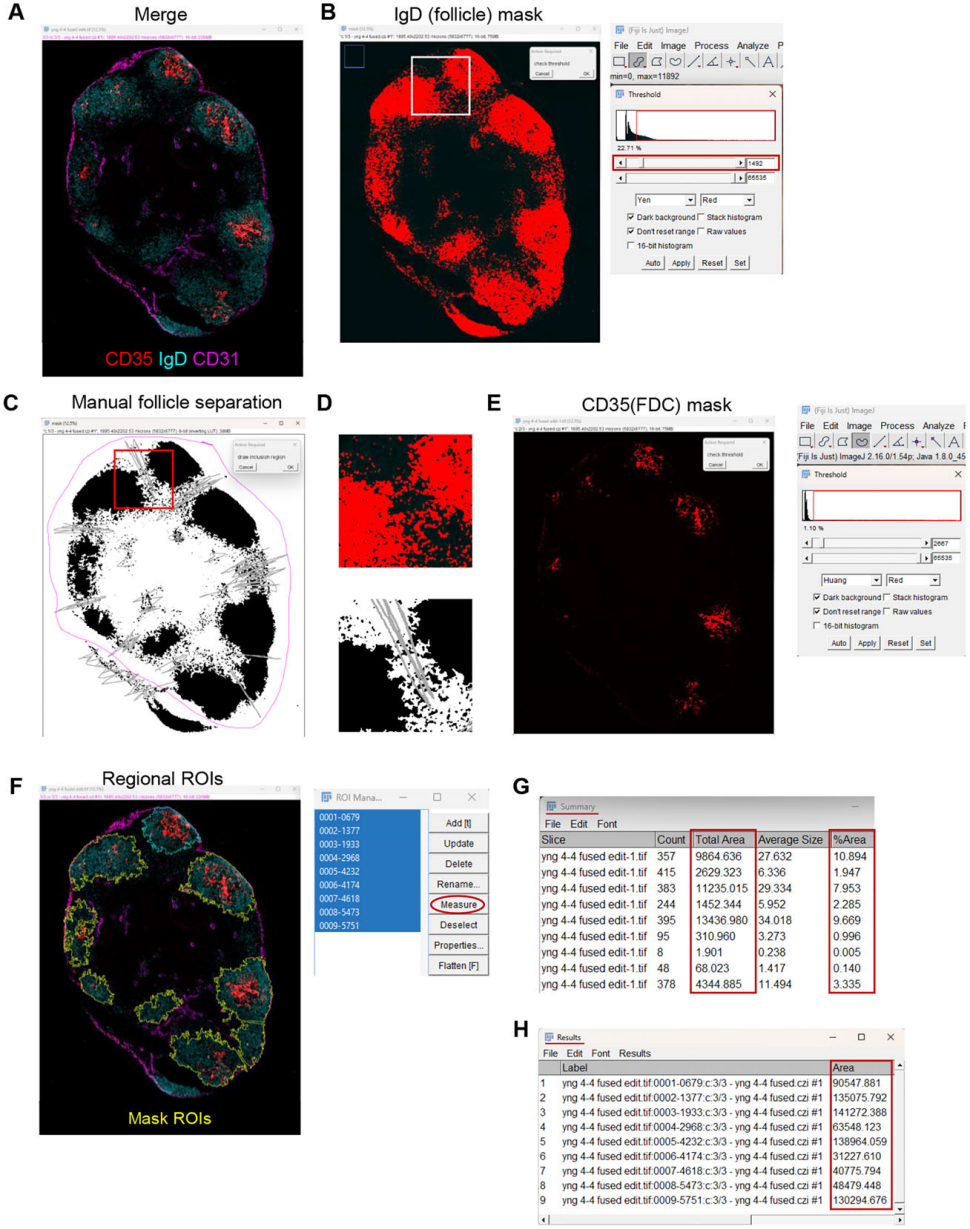
Step‐by‐step visualization of Basic Protocol 2. (**A**) Pre‐processed tiled microscopy image of IF‐stained cervical lymph node using the 20× objective lens, opened in Fiji. Follicles identified by IgD staining in cyan, T cell zone by CD3 staining in red, and endothelium by CD31 staining in magenta. (**B**) Basic Protocol 2, step 3: example of manual thresholding to identify cell follicles. (**C**) Example of processed lymph node image after Basic Protocol, 2 step 5, showing the split follicles in grey and the drawn inclusion region in magenta. (**D**) Inset images from (**B**) and (**C**), highlighting using the paint tool to separate follicles, per Basic Protocol 2, step 4. (**E**) Basic Protocol 2, step 6: example of manual thresholding to identify FDCs. (**F**) Analysis output: ROIs for each individual follicle overlaid onto the analyzed lymph node and ROI manger. (**G**) Analysis table of FDC network area with key measurements highlighted in red. (**H**) Analysis table of individual follicle areas with key measurements highlighted in red. Also see Video 2.

1 Pre‐process images as described in Basic Protocol 1 into TIFF files using Fiji/ImageJ (Fig. 2A).

**Video 2:** Using the KVA4 macro. 00:06 to 00:22: Adjusting threshold settings to detect B cell follicles (Channel 1). 00:23 to 00:45: Separating fused follicles using the “brush” tool. 00:46 to 01:00: Selecting the follicles to be included in the macro analysis (yellow line, “freehand selection” tool). 01:01 to 01:07: Adjusting threshold settings to detect FDC network area (Channel 2). 01:14 to 01:19: Demonstrating the follicle masks on the ROI manager. 01:20 to 01:57: Copying the FDC measurements from the “Summary” table. 01:58 to 02:02: Measuring the follicle mask areas using the ROI manager. 02:03 to 02:59: Organizing the results on Microsoft Excel.

2 Drag‐and‐drop the KVA4 macro file onto the Fiji toolbar, click on the image of interest, then press “run.” KVA4 clears previous selections and ROIs from the ROI manager and previous measurement results, and retrieves the image name, dimensions, and voxel size.
3 KVA4 then duplicates channel 1 of the image to create a mask, corresponding to IgD in this example, which identifies the B cell follicles. To create masks of each follicle, KVA4 first smoothens the image by running “Gaussian Blur” (Process > Filter > Gaussian blur…) with a sigma = 5, then auto‐thresholding (Image > Adjust > Threshold) with the “Yen dark” method. The user will be prompted to manually check the thresholding values to properly visualize the follicles and minimize both background fluorescence and gaps within the follicle area (Fig. 2B). After thresholding, KVA4 converts the channel area into a mask (Process > Binary > Convert to Mask) and fills any holes (Process > Binary > Fill Holes).
4 The user will then be prompted to “split regions;” editing the mask to ensure each follicle is distinct. Select the paintbrush tool and draw lines between the follicles (Fig. 2C, red square; Fig. 2D) guided by the original staining (Fig. 2B, white square). For staining outside of follicles, e.g., around HEVs or in the deep cortical ridge (Takeuchi et al., 2018), use the paintbrush tools to disrupt the thresholded image. Then press “OK.” If the follicles are distinct, ignore this command and press “OK.”
5 The user is next prompted to “draw inclusion area” to exclude any regions of unwanted staining, e.g., staining outside the lymph node. Use the freehand selection tool to outline the area to be included (Fig. 2C, pink outline). This command can be ignored by pressing “OK.” KVA4 will then run the “Analyze Particles” command (Analyze > Analyze Particles…) to create ROIs of each follicle and add them to the ROI manager.
  Note that the follicle measurements are not shown at this stage; see step 8.
6 KVA4 next selects the original image and duplicates Channel 2, which corresponds to CD35 staining to identify FDCs. The image is auto‐thresholded and the user is prompted to manually confirm the thresholding values (Fig. 2E).
7 Finally, KVA4 counts the follicle masks in the ROI manager (Fig. 2F), selects each one, and runs the “Analyze Particles” command to measure the area of FDC staining within each follicle. A new summary window will open, presenting the results (Fig. 2G).
8 To measure the size of each B cell follicle, select all the follicle masks in the ROI manager (Fig. 2F) and click “measure.” This will open a new results window containing the area measurements of each follicle (Fig. 2H).
9 The data should then be exported to data processing software to be collated and analyzed.

## COMMENTARY

### Critical Parameters

The analysis tools presented here aim to automate much of the analysis process, ensuring more consistent and impartial results. There are several points throughout the experiment process where user input is required. To avoid introducing unconscious biases and affecting the integrity of the results, we recommend the following during image acquisition.

All images should be obtained using the same microscope settings. Attention should be paid to optimizing the “gain” and “exposure” to apply across multiple test slides from all experimental groups. Once the appropriate settings to adequately visualize stained tissues are established, they should be applied for subsequent microscopy sessions.

Before using the macros we have described here, users may wish to ensure that they are running the latest version of Fiji. To determine the installation version, run the <help><about ImageJ…> commands. To install the latest version, run the <help><Update…> command.

During image processing, the brightness and contrast settings should be the same for all images with the same staining. In this instance, subtle differences between tissue sections may require some finetuning of the brightness and contrast, although users should be mindful to maintain similar settings between images without affecting the quality of each image. The same principle should apply to all manual thresholding the users will be prompted to do while running the macros. Differences between tissues will require fine adjustments in the exact thresholding settings and users should maintain similar settings for each fluorescence channel across all images of the same stain. It is crucial that users optimize the manual thresholding by trying different settings during their initial attempts, as thresholding will determine how the macros identify the distinct areas of secondary lymphoid organs and create the corresponding masks that will produce the final measurements.

It is important to note that both macros assume each stain has been assigned to a specific channel: IgD on channel 1, CD3 or CD35 on channel 2, and Lyve‐1 on channel 3. This order is primarily determined by the acquisition settings of the microscope software used. When using images with antibodies in a different order, channel order may be re‐assigned during image pre‐processing, using the “Merge channels” command (Image > Color > Merge channels…). Alternatively, the macro script may be adapted accordingly to use different channel orders.

If staining is highly variable, blinding the groups will ensure unbiased results.

### Troubleshooting

The troubleshooting table gives detailed solutions to the most common issues we have encountered using these macros (Table 1).

**Table 1:** Troubleshooting Common Image Analysis Problems

Problem	Possible causes	Solutions
KVA1, lymph node mask inaccurate	Improper thresholding of whole tissue	In Basic Protocol 1, step 10: if only part of the lymph node is included in the mask, increase the thresholding value; if external structures (e.g., fat remnants) or background staining are included in the mask, decrease the thresholding value
KVA1, lymph node area measured inaccurately	Improper thresholding of individual channels	In Basic Protocol 1, step 12: adjust thresholding value for the channels that correspond to the inaccurate area
KVA4, macro “fuses” multiple follicles	Incorrect/insufficient manual separation of tissues	In Basic Protocol 2, steps 3‐4: Adjust thresholding value. Then, ensure proper and clear separation of individual follicles using the paintbrush tool in steps 4‐5 (see Fig. 2C)
KVA4, non‐follicular regions included in follicle mask	Improper selection of inclusion area and/or non‐specific staining artifacts too close to actual follicles	In Basic Protocol 2, steps 4‐5: Ensure inclusion area has been drawn as accurately as possible; to exclude staining outside the follicle, use the paintbrush or eraser tools to disrupt the thresholded image so they are smaller than the minimum size recognized by the “analyze particles” parameters
		If the issue persists, exclude any measurements of inaccurately selected regions from further analysis

### Understanding Results

#### KV1 output

The results of the analysis are presented in a new window, in the order that the macro performs each measurement (Fig. 1E). The first line will always contain the measurements for the whole LN, titled “AVG_” followed by the full name of the image file. The measurements for each channel of the image then follow sequentially.

Area: The total area occupied by the lymph node or each lymphoid compartment in µm^2^. This is determined by the mask ROIs that the macro creates using the manual threshold values input by the user. Note that the measurement unit depends on how the image is acquired and calibrated; in this example, the images have been auto scaled to µm by the acquisition software.

Mean gray value: The average fluorescence intensity of pixels within each mask ROI.

Min/Max gray values: The minimum and maximum fluorescence intensity of pixels within each mask ROI.

%Area: The percentage of the whole lymph node that is occupied by each compartment. Note that the %Area of the whole tissue will instead reflect the percentage of the pixels with any fluorescent signal.

Min/MaxThr: The minimum and maximum thresholding values chosen by the user for each channel. These can be useful for ensuring that similar values are chosen across all LNs belonging to the same group, thus maintaining objectivity, and reducing external variation.

#### KV4 output

When the analysis is complete, KVA4 will present the measurements for the FDC area within each follicle ROI in a new “summary” window (Fig. 2G). Although the area of each ROI is measured for this process, this data is not directly shown. Basic Protocol 2, step 8, describes how to obtain these area measurements from the ROI manager (Fig. 2F), which will then be shown in a separate “results” window (Fig. 2H).

Count: The total count of individual CD35+ particles in each follicle ROI. Note that this does not correspond directly to individual FDCs; this tool is not able to accurately distinguish each cell, rather it counts individual CD35+ structures that fit the analysis parameters.

Total Area: The area of the FDC network in each follicle ROI in µm^2^. Note that the measurement unit depends on how the image is acquired and calibrated; in this example, the images have been auto scaled to µm by the acquisition software.

Average Size: The average CD35+ particle size within each follicle ROI.

%Area: The percentage of each follicle area that is occupied by FDCs.

Mean gray value: The average fluorescence intensity of CD35+ pixels within each follicle mask.

#### Example Data

**Figure 3:**
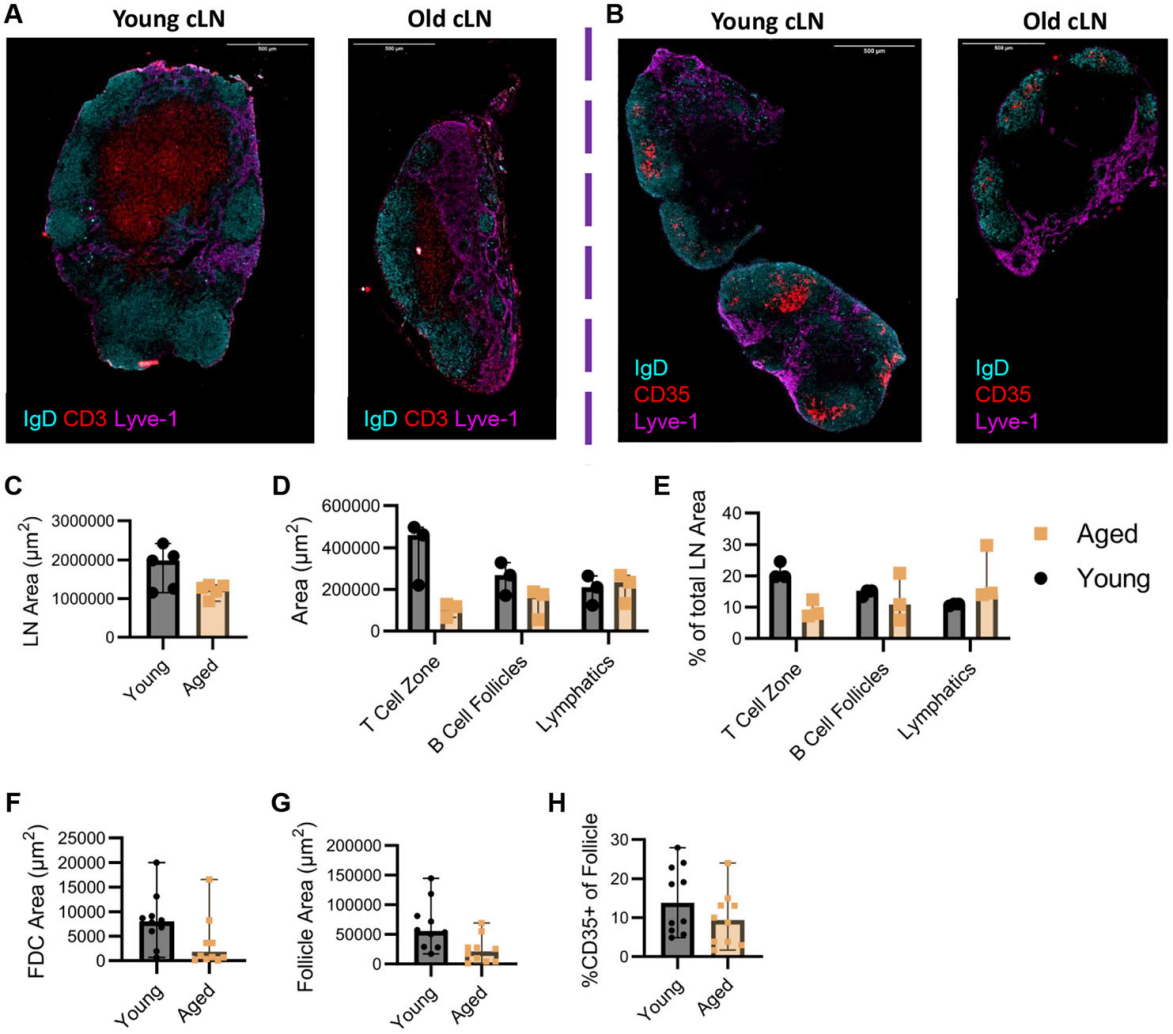
Example data from young and aged cervical lymph nodes. Cervical lymph nodes (cLNs) from young adult and aged mice were stained for IgD (cyan), LYVE‐1 (magenta), and CD3 (red‐A) or CD35 (red‐B) to identify B cell follicles, the LEC network, and the T cell zone or FDC network, respectively. (**A**‐**B**) Representative images of young and old cLNs. Scale bar, 500 µm. (**C**‐**E**) KVA1 analysis of young (5 month) and aged (>22 month) mouse cervical lymph nodes. (**C**) Lymph node area (*n* = 5). (**D**) Area of the T cell zone, follicles, and lymphatics (*n* = 3) (**E**) Proportion of the total lymph node area occupied by the T cell zone, follicles, and lymphatics (*n* = 3). (**F**‐**H**) KVA4 analysis of follicles (*n* = 10) from young (5 month) and aged (>22 month) mouse cervical lymph nodes. (**F**) Area of FDC network in individual follicles. (**G**) Area of individual follicles. (**H**) Proportion of follicle area occupied by the FDC network.

To test whether these macros could detect the well‐established effects of aging on lymphoid tissue architecture (Cakala‐Jakimowicz et al., 2021; Lancaster, 2023), we determined the spatial organization of cervical nodes isolated from younger adult and aged animals (Denton et al., 2022) using KVA1 and KVA4. The lymph node area tends to be smaller in aged mice (Fig. 3A,C), with the T cell zone typically showing a greater reduction in aged lymph nodes compared to other lymph node regions (Fig. 3D‐E), in line with previous studies (Luscieti et al., 1980; Nikolich‐Žugich, 2014). Likewise, analysis of B cell follicles demonstrated the FDC area tends to be reduced in aged lymph nodes (Fig. 3B,F‐H), in agreement with previous studies (Aydar et al., 2003; Denton et al., 2022; Silva‐Cayetano et al., 2023; Szakal et al., 1990; Turner & Mabbott, 2017). Thus, these macros accurately measure lymph node compartmentalization and can be used to automate analysis of lymphoid tissue sections.

### Time Considerations

The exact time required to complete each protocol described here is highly dependent on the number of images to be analyzed, the file size, and the processing power of the computer. Typical processing times are as follows: loading and pre‐processing, 5 to 10 min per image; image analysis using either macro, 5 to 10 min per image; data export, organization, and statistical analysis, 30 to 45 min for the dataset (up to 10 images).

### Author Contributions

**Kassandra Vezyrgianni**: Investigation; methodology; writing—original draft. **Stephen Rothery**: Methodology; writing—review and editing. **Alice E. Denton**: Conceptualization; funding acquisition; supervision; writing—review and editing.

### Conflict of Interest

The authors declare no conflicting interests.

## Data Availability

Macros are available on GitHub page at: https://github.com/DentonLab/LN‐Architecture‐Macros.git
